# Heterogeneous Effects of Health Insurance on Rural Children’s Health in China: A Causal Machine Learning Approach

**DOI:** 10.3390/ijerph18189616

**Published:** 2021-09-12

**Authors:** Hua Chen, Jianing Xing, Xiaoxu Yang, Kai Zhan

**Affiliations:** 1 School of Insurance, Central University of Finance and Economics, Beijing 102206, China; huachen@cufe.edu.cn (H.C.); 2020210863@email.cufe.edu.cn (J.X.); 2021210758@email.cufe.edu.cn (X.Y.); 2 School of Finance, Guangdong University of Foreign Studies, Guangzhou 510410, China

**Keywords:** health insurance, children’s health, causal forest, heterogeneous treatment effects

## Abstract

This paper investigates the impact of Urban and Rural Resident Basic Medical Insurance (URRBMI) on the health of preschool and school-age children in rural China using data from the 2018 wave of the China Family Panel Studies (CFPS). We employ the propensity score matching approach and causal forest to evaluate impacts. Results show that the URRBMI has significantly improved the health status of preschool children. However, the health improvement of school-age children by URRBMI is only limited to obese children, and this effect is not significant. In addition, this paper identifies important variables related to heterogeneity through the causal forest and evaluates the heterogeneity of the impact of URRBMI on the health of two types of children. For preschool children, we find disadvantaged mothers (i.e., with lower wealth, lower educated, or in rural areas) benefit more from the URRBMI. No significant heterogeneity is found for the school-age children. Our study demonstrates the power of causal forest to uncover the heterogeneity in policy evaluation, hence providing policymakers with valuable information for policy design.

## 1. Introduction

Medical insurance is one of the most important policies to guarantee residents’ health and ensure stable social development. Policymakers around the world are committed to expanding the coverage of medical insurance and improving the level of medical insurance through medical reform. Since the late 1990s, the Chinese government has continuously reformed and improved the medical security system. China established Urban Employee Medical Insurance in 1998. China also piloted the New Cooperative Medical Scheme (NCMS) and Urban Resident Basic Medical Insurance (URBMI) in 2003 and 2007, respectively. NCMS and URBMI were integrated into the Urban and Rural Resident Basic Medical Insurance system (URRBMI) in 2016. According to data from the National Medical Insurance Administration of China, by 2020, medical security had achieved universal coverage in China. Besides, data (data from the National Healthcare Security Administration http://www.nhsa.gov.cn, accessed on 8 June 2021) shows that the funding criteria and subsidy standard of URRBMI in most areas have also increased year by year. URRBMI has become an important part of the medical security system for rural residents. It covers more than 800 million rural people and the participation rate of the rural poor population is 99.8%.

Accordingly, the question of “Does Urban and Rural Resident Basic Medical Insurance effectively improve the health status of the rural residents?” has attracted the attention of many scholars and policymakers. Many scholars have conducted multi-angle research on this issue in recent years. For example, Lei and Lin use “Have you been sick in the past four weeks” and “Self-rated health” to measure an individual’s health condition and have found that medical insurance improves the health level of participants as a whole [1]. Cheng et al. found that the NCMS has significantly improved the daily living and cognitive function of the elderly in rural areas, but it has no significant effect on the mortality rate of the elderly within three years [2]. Sun found that the role of URRBMI in improving health status and reducing medical expenditure is limited [3]. Shao found that National Health Insurance has only had an effect on the death rates, and it is the least healthy who benefit the most [4].

From the literature above, we can find that most of the existing studies focus on the elderly in rural areas, because the elderly have a higher risk of illness. However, children are also at a high risk of illness, and their medical insurance has not attracted enough attention in China. In China, children in rural areas are covered by URRBMI, just like adults. While the US, Vietnam, and other countries have social medical insurance specifically designed for children. Children have different physical characteristics, disease risk, and family resource allocation than adults. Therefore, the impact of URRBMI on children may be different from that of adults. However, no research systematically assesses the impact of URRBMI on children’s health.

This paper uses the data from the China Family Panel Studies (CFPS) in 2018 and employs the Propensity Score Matching (PSM) approach to estimate the causal effect between URRBMI and children’s health status. We examine the impact of rural children’s participation in URRBMI on “Height by Age Z-score (HAZ)”, “Weight for Age Z-score (WAZ)”, “Weight for Height Z-score (WHZ)”, and “Body Mass Index (BMI)”. Due to the significant differences in geography and economic development levels between different regions in China, we also consider the heterogeneity of the effects of the URRBMI. The analysis of the heterogeneity is helpful for policymakers to understand the differences in the effect of URRBMI on different groups, and to propose targeted policies for different groups, as well as maximize the effect of URRBMI.

In traditional econometrics, there are two common methods for evaluating the heterogeneity of treatment effects. One method is subgroup analysis. Petticrew et al. pointed out that subgroup analysis may lead to invalid inferences [5]. Another method is multiplicative interaction models. Hainmueller and Mummolo found that the results based on the interaction are fragile and model-dependent [6]. Neither of these two methods can investigate the heterogeneity of the data systematically and comprehensively, which may lead to the omission of important conclusions.

Thus, we use the “Causal Forest”, a method based on random forest and proposed by Athey et al., to estimate the heterogeneity of the treatment effect [7]. This method combines traditional non-parametric methods with machine learning, and it can capture how the treatment effect changes with the changes in the value of the covariates flexibly, i.e., the Conditional Average Treatment Effect (CATE). Compared with the traditional method, this method has two advantages. First, it makes up for the shortcomings of the traditional method. There is also no need to make assumptions about the parameters in the regression. Second, it can use algorithms to capture the CATE of previously unknown subgroups.

The contribution of this paper has the following two aspects. Firstly, we estimate the impact of URRBMI on the health status of children in rural China through the PSM approach. We also use the causal forest to analyze the heterogeneity of the effects and identified the driving factors that cause the heterogeneous effects. Secondly, this article combines the research on policy effects with machine learning. Compared with econometric methods, the causal forest performs better when analyzing heterogeneous effects. It can be used not only to estimate the heterogeneity of URRBMI but also to predict various macro indicators and evaluate policy effects, such as the effect of labor market reform, the effect of tax system reform, etc.

The remainder of this paper is organized as follows. In Section 2, we introduce the background of URRBMI and give a literature review. Section 3 describes the dataset and descriptive statistics. Section 4 presents the empirical framework. Section 5 shows the results. Section 6 concludes and provides recommendations.

## 2. Background

### 2.1. The Urban and Rural Resident Basic Medical Insurance (URRBMI)

The Chinese government attaches great importance to the construction of the medical security system. During the period of the centrally planned economy, China established a free medical system and medical labor insurance covering urban areas, as well as the traditional Cooperative Medical System (CMS) covering rural areas. Relevant data (data comes from the National Healthcare Security Administration http://www.nhsa.gov.cn, accessed on 8 June 2021) shows that in 1978 when the traditional rural Cooperative Medical System was in its heyday, it protected 90% of farmers. After 1978, China conducted market-oriented economic reforms, and the rural people’s communes disintegrated. As a result, the CMS lost its economic foundation and gradually collapsed. By 1993, the coverage rate of medical insurance in rural areas had fallen from 90% to less than 10%. Due to limited insurance coverage and rising medical expenses, many rural families had fallen into or relapsed into poverty. To solve these problems, the Chinese government launched a mutual system of medical assistance and aid for rural areas in 2003, i.e., the NCMS, which focuses on the overall planning of major illnesses. The NCMS and URBMI were integrated into the Urban and Rural Resident Basic Medical Insurance system (URRBMI) in 2016. After more than ten years of development, rural families across China have received varying degrees of medical protection through URRBMI. Before the integration, the enrollment of URRBMI was voluntary, but it had to be as a family unit, and the eligibility for participation was determined by the household registration, which is different from universal health insurance in most developed countries. In addition, China’s hukou system restricts the movement of households across counties, thereby reducing potential spillover effects. The funds for the URRBMI come from individual contributions and subsidies from the central and local governments.

### 2.2. Literature Review

Many scholars have studied the impact of children’s participation in medical insurance from different perspectives. Hanratty estimates the impact of national health insurance on infant health by exploiting the variation across provinces in dates of implementation [8]. Currie et al. point out that the expansion of the Medicaid program in the United States has improved mothers’ access to prenatal care and children’s access to preventive care, and reduced the incidence of low birth weight and infant mortality. However, there is no positive effect on the health status of older children [9,10,11,12]; Wagstaff and Pradhan study the impact of Vietnam’s national health insurance on children’s health and medical utilization [13]; Chou found that the national health insurance plan in Taiwan can effectively reduce infant mortality [14]; while Chen and Jin found that the NCMS does not affect the mortality of pregnant women and young children in rural areas [15]; Alcaraz C found that public health insurance in Mexico affects children’s school attendance and academic performance [16]; Lisa Bagnoli pointed out that the positive impact of health insurance is concentrated among the lower-income households in regions with a high quality of public health care [17].

In empirical research, compared with traditional econometric methods, machine learning methods have more advantages when analyzing heterogeneity effects. Many scholars have researched this issue in recent years. Athey and Imbens first proposed a method based on regression trees, called “Causal Tree”. This method relies on a simple division of samples. Half of the samples are used to construct the tree structure, and half of the samples are used to estimate the treatment effect [7]. However, the disadvantage of this method is that the estimates of the causal trees do not represent individual treatment effects. Subsequently, Athey and Imbens propose “Causal Forest” to estimate the heterogeneity of treatment effects based on random forest. The causal forest makes up for the shortcomings of the causal tree because it can capture the treatment effects of individuals [18]. In addition, Wager and Athey prove that the causal forest can capture the heterogeneous conclusions missed by traditional methods [19].

At present, there is little literature using the causal forest to estimate the treatment effect. Chin estimates the impact of the opening of the new subway on the surrounding apartments based on multivariate hedonic methods and causal forest respectively [20]. Andrew used the causal forest to estimate the negative effects of the financial crisis on economic growth [21]. Noemi et al. used the data from the Indonesian Family Life Survey to identify the groups that benefit most from national health insurance through the causal forest [22].

## 3. Data and Descriptive Statistics

### 3.1. Data and Variables

The data in this paper comes from the China Family Panel Studies (CFPS). The CFPS is a national and comprehensive social tracking survey implemented by the Institute of Social Science Survey (ISSS) of Peking University. The CFPS aims to collect data at the individual, family, and community levels to reflect the changes in China’s society, economy, population, education, and health. It covers 25 provinces, municipalities, and autonomous regions in China. The CFPS conducted a baseline survey in 2010 and a full sample follow-up survey every two years thereafter. We used the data of 2018 in this paper because the latest data from 2020 has not been published.

The sample in this paper comes from the Children’s Database in 2018. To isolate the impact of the URRBMI on children’s health, we excluded children who participated in other social medical insurance and commercial medical insurance. After excluding samples with errors and missing information, we had 5552 samples in total. We define the treatment group as “Rural children participating in URRBMI in 2018” and the control group as “Rural children without any medical insurance in 2018”. There were 4760 samples in the treatment group and 792 samples in the control group.

The independent variable in this paper is URRBMI, which represents “Does the child participates in the URRBMI or not”, the value of participants was 1, and the value of non-participants was 0. The dependent variable is the health status of the child. At present, WHZ, WAZ, and HAZ are commonly used internationally to measure the health of preschool children (0 to 5 years old). We used the above three indicators to estimate the short-term, mid-term, and long-term health levels of preschool children. As for school-age children (6 to 16 years old), we used the child’s Body Mass Index (BMI) to measure their health. BMI can reflect the obesity and health status of children. Since China is a developing country, the rural population is relatively poor, and malnutrition rather than obesity is more common among rural children. Therefore, the increase in BMI can be seen as evidence of the improvement of children’s health. In addition, studies have shown that the health of children will be affected by factors from the family, social and economic environments. For example, the mother’s age and education level, the family’s per capita income, and the availability of medical resources. Therefore, we controlled these factors in our research.

### 3.2. Descriptive Statistics

Table 1 shows the definition and descriptive statistics of variables. We compared the differences in sample proportions between the treatment group and the control group by the t-test and found that the two groups had significant differences in the WAZ, WHZ, and BMI. Since the WHZ and WAZ reflect the health status of children in the short-term and medium-term, respectively, the significant difference between the two groups indicates that for preschool children, the short-term and medium-term health status of children insured by URRBMI was significantly higher than that of children uninsured by URRBMI. The BMI reflects the degree of body fatness and nutritional status. The BMI of insured children was 18.283, which is lower than the normal range, indicating that there is a state of malnutrition among these children. The BMI index of uninsured children was higher than that of insured children, which may be due to the higher family economic level of uninsured children. In addition, compared with uninsured children, insured children were older, their mothers were older and less educated, and most of them lived in economically underdeveloped areas in the central and western regions, which is consistent with the fact that the per capita income of insured children’s families is lower.

## 4. Empirical Framework

### 4.1. Theoretical Basis

This article mainly estimates the Average Treatment Effect (ATE) of the URRBMI on the health status of children in the treatment group. In the framework of potential results proposed by Neyman and Rubin, each individual has two states: “Participating in the URRBMI” and “Not participating in the URRBMI”. Each state corresponds to a potential outcome. $T_{i}$ indicates the treatment effect, $T_{i}=1\;$ indicates that the individual has participated in the URRBMI, and $T_{i}=0$ indicates that the individual has not participated in the URRBMI. $y_{1i}$ refers to the potential results when participating in the URRBMI, and $y_{0i}$ refers to the potential results when not participating in the URRBMI. Based on the assumptions above, the treatment effect of URRBMI on individuals is the difference of potential results in different states, i.e., $\alpha_{i}=y_{1i}-y_{0i}$. Therefore, the Average Treatment Effect (ATE) of URRBMI is $\alpha_{ATE}=E\left[y_{1i}]-E[y_{0i}]=E[\alpha_{i}\right]$. The Average Treatment Effect on the treated (ATT), i.e., insured children is $\alpha_{ATT}=E\left[y_{1i}|T_{i}=1\right]-E\left[y_{0i}|T_{i}=1\right]=E\left[\alpha_{i}|T_{i}=1\right]$. The Average Treatment Effect on the control (ATC), i.e., uninsured children is $\alpha_{ATC}=E\left[y_{1i}|T_{i}=0\right]-E\left[y_{0i}|T_{i}=0\right]=E\left[\alpha_{i}|T_{i}=0\right]$. However, under normal conditions, an individual has only one state. For the insured children, we could only observe the outcomes when they participated in URRBMI; For the uninsured children, we could only observe the outcomes when they did not participate in URRBMI. Therefore, we could not get the ATE, ATT, and ATC. In this case, if we assumed that an individual satisfies the Conditional Independence Assumption (CIA), that is, the allocation of individual states and potential results are independent of each other, then $y_{1i},y_{0i}⊥T_{i}|X_{i}$, $E\left[y_{0i}|T_{i}=1\right]=E\left[y_{0i}|T_{i}=0\right]$, $E\left[y_{1i}|T_{i}=1\right]=E\left[y_{1i}|T_{i}=0\right]$, We could use the observation result $E\left[y_{0i}|T_{i}=0\right]$ of the control group to replace the counterfactual result $E\left[y_{0i}|T_{i}=1\right]\;$ of the treatment group to obtain the Average Treatment Effect on the treated (ATT).

### 4.2. Propensity Score Matching (PSM)

Since participation in the URRBMI is a voluntary choice, the unobserved heterogeneity between insured and uninsured children may lead to selection bias. We used the Propensity Score Matching (PSM) to eliminate the influence of selection bias on the estimation results.

There are two prerequisites for use of PSM: (1) the conditional independence assumption. (2) the common interval assumption. Let P(x)=Prob(T=1|x) be the propensity index, the common interval requires 0<Prob(T=1|x)<1. Under these two assumptions, Rosenbaum and Rubin prove that $y_{1},y_{0}⊥T|P\left(x\right)$, 0<Prob(T=1|p(x))<1 [23].

We matched the samples under the two assumptions:

1. Define similarity: This stage included two parts. First, we selected confounding factors that affected both the independent variable and the dependent variable as covariates based on the Conditional Independence Assumption. These covariates were used to define similarity. The covariates included the child’s gender and age, mother’s age and education, household income per capita, and family’s living area and geographic location. Secondly, we needed a measure to define similarity. Generally, Euclidean distance and Mahalanobis distance were used. We used Euclidean distance here.

2. Implement matching: We estimated the propensity index through logit regression, and the regression equation is:

(1) $$HI_{i}=\beta_{0}+\beta_{1}X_{Ci}+\beta_{2}X_{Mi}+\beta_{3}X_{Hi}+u_{i}$$

In Equation (1), $HI_{i}$ indicates whether the child participates in the URRBMI or not. $X_{Ci}$ represents the child’s characteristics. $X_{Mi}$ represents the mother’s characteristics, including age and education. $X_{Hi}$ represents the family characteristics, including household income per capita. We used Euclidean distance to estimate the propensity index and implement matching. The matching methods included nearest-neighbor matching, kernel matching, local linear regression matching, and spline matching. In this paper, we used one-to-many neighbor matching. For each individual in the treatment group, we found multiple individuals in the control group to match with them. Se also used kernel matching.

3. Treatment effect estimation: We used Equation (2) to estimate the ATT based on matched samples.

(2) $$\alpha_{ATT}=E_{p|T=1}\left\{E\left[y_{1}|T=1,p\left(x\right)=p\right]-E\left[y_{0}|T=0,p\left(x\right)=p\right]\right\}$$

### 4.3. Causal Forest

The estimates of ATE, ATT, and ATC represented three different treatment effects, but it could not capture how the treatment effect changed with changes in the covariates. To estimate the difference of treatment effects in different subgroups, we defined the Conditional Average Treatment Effect (CATE) as:

(3) $$\tau \left(x\right)=E[y_{1i}-y_{0i}|X=x]$$

CATE is equivalent to a function of covariates X. Different values of covariates corresponded to different subgroups. Then we could get the average treatment effect of different subgroups. We defined the partially linear model of the dependent variable $Y_{i}$ as:

(4) $$Y_{i}=f\left(X_{i}\right)+W\tau +\varepsilon_{i}$$

$f\left(X_{i}\right)$ is a function of covariates X. According to Robinson [24], we rewrote Equation (4) as a form of residual:

(5) $$Y_{i}-m\left(X_{i}\right)=\left(W-p\left(X_{i}\right)\right)\tau +\varepsilon_{i}$$

In Equation (5), $p\left(X_{i}\right)$ represents the propensity index, $m\left(X_{i}\right)=E\left[Y_{i}|X_{i}\right]$ represents the conditional expectation of $Y_{i}$ under the given covariate X. $p\left(X_{i}\right)$ and $m\left(X_{i}\right)$ can be estimated by any prediction algorithm including the ML method. Substituting $p\left(X_{i}\right)$ and $m\left(X_{i}\right)$ into the regression result of (5), and assuming that there was a sufficiently small neighborhood N(x) to make τ(x) constant in the neighborhood, we obtained the estimator of the average treatment effect τ:

(6) $$\hat{\tau }=\frac{\sum_{\left\{i:X_{i}\in N\left(x\right)\right\}}\left\{\left(W_{i}-\hat{p}\left(X_{i}\right)\right)\left(Y_{i}-\hat{m}\left(X_{i}\right)\right)\right\}}{\sum_{\left\{i:X_{i}\in N\left(x\right)\right\}}{\left\{\left(W_{i}-\hat{p}\left(X_{i}\right)\right)\right\}}^{2}}$$

Such a form decreased the sensitivity of the resulting estimator to the errors in the estimates of the $p\left(X_{i}\right)$ and $m\left(X_{i}\right)$ [25]. The choice of N(x) is essential to estimating CATE. In this regard, Athey et al. proposed the causal forest [26], which regards N(x) as a locally weighted set of adjacent values under the specific x. In the random forest [27], we selected the covariate and the critical value of this covariate at each node. We minimized the residual squares of the two child nodes and grouped the samples so that the samples in the same group were very similar in covariates, and the samples in different groups had the largest difference in the value of the dependent variable. The above process formed a “decision tree”. Groups with similar covariates were defined as the “leaf” of the tree, and the average value of the dependent variable of all samples in the same leaf was the predicted value of these samples.

To reduce the prediction error of a single tree, we used bootstrap to generate different trees and average the estimates of all trees [28,29], through which we reduced the variance of the estimator and improved the prediction accuracy of the model.

To estimate the heterogeneity of the treatment effect, the causal forest was improved from the following aspects based on the random forest: Firstly, the causal forest introduced “honesty trees”. The specific method was to divide the sample data into two sub-samples: one was used to generate a causal tree, and the other was used to estimate the average treatment effect of the tree. In this way, different information was used for the selection and estimation of the model, and the bias caused by using the same information was reduced. Secondly, the causal tree in the causal forest was different from the decision tree in the random forest. Each “leaf” of the causal tree contained individuals from the treatment group and control group. So each “leaf” constructed the counterfactual of the treatment group, and the average treatment effect of the “leaf” could be directly obtained. Therefore, the segmentation criterion when constructing the causal tree was no longer the value of the covariate in the random forest, but the average treatment effect. We needed to ensure that the individuals in the same group were as similar as possible, and the difference in treatment effects between different groups was maximized. Ultimately, the average treatment effect within a group was the prediction of the treatment effect in this group.

In addition, the causal forest did not directly average the estimates of all trees but used the forest obtained to estimate the weighting function. Additionally, it used the weighting function to modify the CATE:

(7) $$\hat{\tau }=\frac{\sum_{i=1}^{n}\alpha_{i}\left(x\right)\left\{\left(W_{i}-\hat{p}\left(X_{i}\right)\right)\left(Y_{i}-\hat{m}\left(X_{i}\right)\right)\right\}}{\sum_{i=1}^{n}\alpha_{i}\left(x\right){\left\{\left(W_{i}-\hat{p}\left(X_{i}\right)\right)\right\}}^{2}}$$

The specific steps of the causal forest were as follows [30,31,32,33]:

1. Estimate $m\left(X_{i}\right)$ and $p\left(X_{i}\right)$ through the “regression” forest, and import the estimation into $\hat{\tau }$. We chose 500 trees to adjust the parameters and 1000 trees to estimate the result.

2. Use the bootstrap method to generate a causal forest with 1000 samples. Sort the covariates according to the importance of the covariates in the process of generating the causal forest, and select the covariates whose importance is higher than the average variable.

3. Use the variables selected in step 2 to regenerate the causal forest. Select 500 trees to adjust the parameters, and select 3000 trees to predict the individual treatment effect.

4. Draw the histogram of the individual treatment effect, and give the confidence interval of the average treatment effect. Then, judge whether the result is heterogeneous.

5. Sort the covariates based on their contribution to heterogeneity, and select the variables with high importance to form the new subgroup. Then compare the treatment effects of different subgroups.

We used the causal forest to estimate the heterogeneous effects of URRBMI on the nutritional and health status of preschool children (0–5 years old) and school-age children (6–16 years) in rural areas.

## 5. Results

### 5.1. The Average Treatment Effect

**Figure 1:**
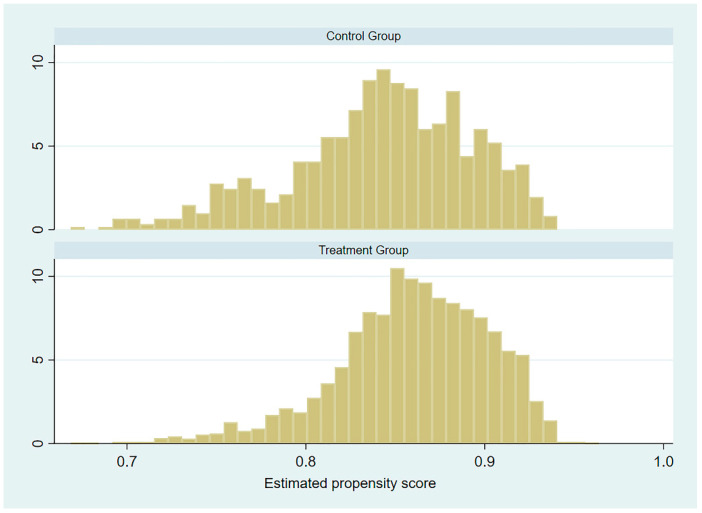
Propensity score histograms.

Figure 1 shows the histogram of the estimates of the propensity index of the treatment group and the control group before matching. Most observations were within the common value range, so propensity score matching could be performed in the common support area.

Table 2 shows the estimates of URRBMI on children’s health using propensity score matching. Columns (3) to (5) give the estimates of one-to-one nearest neighbor matching, one-to-four nearest neighbor matching, and kernel matching with a bandwidth of 0.06 respectively. The coefficients of the three matching methods were slightly different, but the estimates were generally consistent. Thus, the estimation results were relatively robust. We mainly focused on the estimates of kernel matching. It could be seen from columns (5) that for preschool children aged 0 to 5 years, the estimates of WHZ were significant at the level of 1%, and the estimates of WAZ and HAZ were significant at the level of 5%, which indicated that URRBMI has significantly improved the nutritional and health status of insured children in the short-term, medium-term, and long-term. Besides, BMI decreased by an average of 0.687 for the insured school-age children. Therefore, for obese children, participating in URRBMI can reduce the risk of obesity to a certain extent, but this effect was not significant.

The reason why URRBMI has not significantly improved school-age children’s health may come from three aspects: first, we used BMI to measure school-age children’s health in this paper. Internationally, it is generally believed that BMI can reflect age characteristics and is one of the better indicators to evaluate total body fat and measure the nutritional and health status of adolescents. However, there is no appropriate health measurement indicator for school-age children. Therefore, the existing health measurement indicators can not fully reflect the health status of children, which can be further studied when there are more perfect children’s health measurement indicators in the future. Secondly, URRBMI is a system focusing on the overall planning of major illnesses, it intends to reduce the financial burden on the family caused by major illnesses through hospitalization reimbursement. However, its protection for minor illnesses and outpatient reimbursement are relatively inadequate. Many diseases are not covered by URRBMI. Thirdly, compared with preschool children, school-age children have stronger immune systems, high resistance, and a lower risk of serious diseases. Therefore, URRBMI has no significant impact on the health of school-age children.

### 5.2. Heterogeneity Analysis

**Figure 2:**
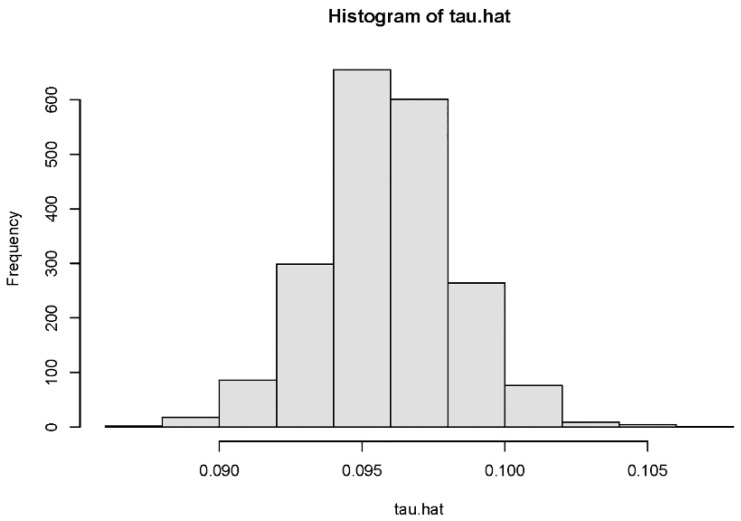
Estimated individual treatment effects of preschool children.

**Figure 3:**
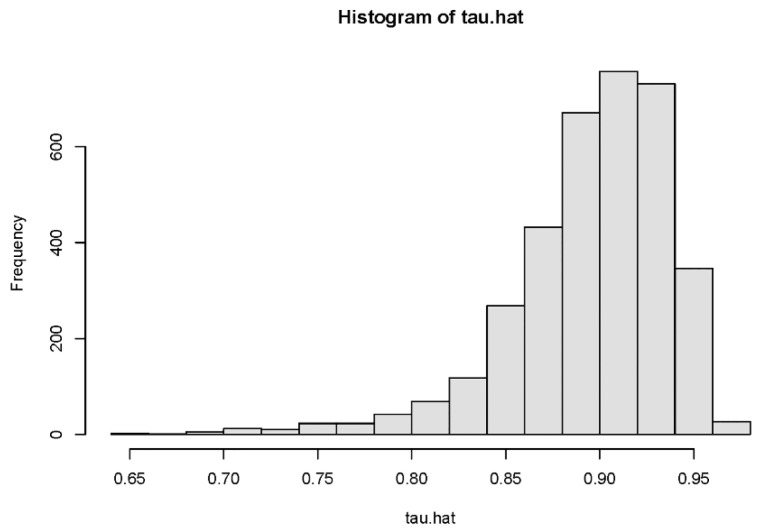
Estimated individual treatment effects of school-age children.

Figure 2 and Figure 3 are histograms of the distribution of conditional individual treatment effects (CITE) for preschool children and school-age children. Figure 2 represents preschool children, and Figure 3 represents school-age children. For preschool children, the 95% confidence interval of the distribution was [−0.042, 0.204], which contains 0. For school-age children, the 95% confidence interval of the distribution was [−2.004, −0.102], which does not contain 0. The heterogeneity analysis of the treatment effect showed that the impact of URRBMI on preschool children was heterogeneous, but there was no heterogeneity in the health impact of URRBMI on school-age children.

Table 3 lists the order of importance of the covariates that affect the treatment effect of heterogeneity. These covariates overlap with the pre-specified socioeconomic covariates to a large extent, i.e., age, education, wealth quintile, and family residential area. For preschool children, the most influential variable was the age of the mother, followed by the age of the child and the education level of the mother. For school-age children, the most influential variable was the mothers’ education level. The physical condition of children aged 0–5 years was more likely to be affected by the physical condition of their mother. The fetus conceived by the young woman will have a lower probability of deformities and dementias. As children grow older, children’s health is more affected by acquired factors such as social and economic conditions. Mothers with a higher education level do a better job in guiding their children to form good living habits, which have a long-term effect on their children’s health.

Table 4 shows the CITE for subgroups discovered by the causal forest. It can be found from Table 4 that there were differences in the ATC of different subgroups. There were strong gradients of ATC in different levels of wealth, different levels of education, and in rural and urban areas. For preschool children, all the differences were significant. While for school-age children, there were differences in ATC in different subgroups, but the differences were not significant. Specifically, for pre-school children, participating in URRBMI will significantly improve the health of children in rural areas by 0.118, and improve the health of children whose mothers have an elementary school education by 0.214. Participating in URRBMI significantly improved the health of male children by 0.098. For school-age children, only children whose families lived in urban areas had significantly increased their BMI by 0.285 after participating in the URRBMI. Participating in URRBMI decreased the BMI of children in other subgroups, but this effect was not significant. Finally, we divided the sample into two groups according to the estimation of CITE: one group was greater than the median, and the other group was less than the median. We compared the characteristics of the covariates of the two groups and found that for preschool children, those with older mothers and lower levels of education, and low family wealth benefited most from the URRBMI. While school-age children whose mothers are older and less educated and living in western rural areas had a greater decline in BMI. We also found that the BMI of children whose mothers have a high level of education, and whose family is wealthier and living in the eastern region will rise, but the impact was not significant.

## 6. Conclusions

This article uses data from CPFS 2018 and takes the HAZ, WAZ, WHZ, and BMI as health indicators to estimate the impact of the URRBMI on the health of preschool and school-age children in rural areas based on previous research. The results show that URRBMI significantly improves the HAZ, WAZ, and WHZ of preschool children, indicating that URRBMI has effectively improved the health status of preschool children. However, the health improvement of school-age children by URRBMI is only limited to obese children, and this effect is not significant. In addition, this paper identifies important variables related to heterogeneity through the causal forest and evaluates the heterogeneity of the impact of URRBMI on the health of two types of children. The results of the causal forest show that the mother’s age and education cause the heterogeneity of treatment effects of preschool and school-age children. The estimation results of different subgroups and the individual treatment effects of samples show that the impact of URRBMI on children’s health varies with socioeconomic status. In particular, URRBMI has a significant positive impact on the health status of preschool children whose mothers’ education level and family wealth level are low. For school-age children whose mothers have low education and live in western rural areas, URRBMI will reduce their BMI. URRBMI will also improve the BMI of children whose mothers have a high level of education and live in a wealthier family in eastern urban areas. However, those impacts were not significant, which shows that URRBMI helps to reduce the health gap of children to a certain extent.

This study is the first to use the causal forest to estimate the heterogeneity of the impact of URRBMI on rural children’s health. We emphasize the role of causal forests in exploring the heterogeneity of policy impacts based on the observed covariates and use the importance of variables to find the main variables causing heterogeneity. This method changes the policy impact assessment from “whether the policy is effective” to “who the policy is effective for”. So, our result has reference value for policymakers to formulate “optimal policy rules” and improve the qualification and subsidy standards of medical insurance. In addition, the causal forest can also be combined with other traditional econometric methods, such as the instrumental variable method. The monotonicity assumption of parameter estimation can be relaxed in the first stage, and the results at different levels can be identified through heterogeneity treatment effect with the help of the causal forest. In the future, causal forests can be used to improve the evaluation of policies based on traditional methods.

This paper also has some shortcomings. First of all, there is no appropriate health measurement standard for school-age children. It is generally accepted that BMI is one of the indicators reflecting age characteristics and health and nutritional status. Thus, the estimation of the impact of URRBMI on the health outcomes of school-age children may be biased. Secondly, although Propensity Score Matching and causal forest help to control observable and unobservable factors, there may still be potential for confounding effects caused by unobservable time-varying factors.

Finally, our research provides empirical evidence for the health effects of URRBMI, which may have important policy significance for the reform of the medical system in China and the development of medical insurance in other developing countries. The health effects of URRBMI in the general population and disadvantaged population deserve further study.

## Figures and Tables

**Table 1:** Descriptive Statistics.

Variables	Definition	Treatment Group	Control Group	Differences
Weight for Height Z-score (WHZ)	WHZ compares a child’s weight to the weight of a child of the same length/height and sex to classify nutritional status.	−0.185 (0.802)	−0.376 (0.770)	−0.191 ***
Weight for Age Z-score (WAZ)	WAZ compares a child’s weight to the weight of a child of the same age and sex to classify nutritional status.	0.022 (1.012)	−0.091 (0.929)	−0.112 **
Height by Age Z-score (HAZ)	HAZ compares a child’s height to the height of a child of the same age and sex to classify nutritional status.	0.020 (0.991)	−0.069 (1.012)	−0.090
Body Mass Index (BMI)	Weight (kg)/height^2^ (m^2^)	18.283 (5.209)	18.944 (6.703)	0.661 **
**Child’s characteristics**				
Gender	1 if male; 0 if female	0.539 (0.499)	0.501 (0.500)	−0.037 *
Age	Age of the child	7.813 (4.241)	5.765 (4.807)	−2.048 ***
**Mother’s characteristics**				
Age	Age of the child’s mother	34.211 (6.590)	32.694 (6.895)	−1.517 ***
Education	1 if no formal education; 2 if primary school; 3 if junior high school; 4 if high school; 5 if junior college; 6 if bachelor degree; 7 if master.	2.676 (1.143)	3.057 (1.393)	0.381 ***
**Family characteristics**				
Household income per capita	Logarithm of household income per capita	8.994 (0.890)	9.079 (1.059)	0.085 **
Living area	1 if urban area; 0 if rural area	0.332 (0.471)	0.455 (0.498)	0.123 ***
Geographic location	1 if eastern region; 2 if central region; 3 if western region	2.047 (0.819)	1.816 (0.827)	−0.232 ***

Note: *, **, and *** indicate that the coefficients significantly differ from 0 at the 10%, 5%, and 1% levels, respectively. The deposit is scaled by its logarithmic form.

**Table 2:** Propensity Score Matching (PSM) estimates of the Urban and Rural Resident Basic Medical Insurance (URRBMI) impact.

Dependent Variables	*N*	Nearest Neighbor Matching (1:1)	Nearest Neighbor Matching (1:4)	Nuclear Match
Weight for Height Z-score (WHZ)	2015	0.199 *** (0.055)	0.183 *** (0.046)	0.189 *** (0.044)
Weight for Age Z-score (WAZ)	2015	0.155 ** (0.064)	0.158 ** (0.056)	0.131 ** (0.053)
Height by Age Z-score (HAZ)	2015	0.213 ** (0.072)	0.168 ** (0.060)	0.134 ** (0.057)
Body Mass Index (BMI)	3537	−0.799 (0.468)	−0.634 (0.396)	−0.687 (0.375)

Note: **, and *** indicate that the coefficients significantly differ from 0 at the 5%, and 1% levels.

**Table 3:** Covariate importance in explaining treatment effect heterogeneity.

Rank	Preschool Children		School-Age Children	
	Variable	Importance	Variable	Importance
1	Age of the child’s mother	0.368	Mother’s education: no formal education	0.263
2	Age of the child	0.158	Age of the child’s mother	0.200
3	Mother’s education: no formal education	0.155	Age of the child	0.149
4	Mother’s education: bachelor degree	0.060	Wealth quantile 5	0.115
5	Geographic location: central region	0.036	Living area: rural area	0.099
6	Mother’s education: primary school	0.033	Geographic location: central region	0.036
7	Child’s gender: male	0.031	Child’s gender: male	0.029
8	Wealth quantile 3	0.025	Geographic location: western region	0.026
9	Living area: rural area	0.022	Mother’s education: junior college	0.019
10	Wealth quantile 2	0.021	Wealth quantile 3	0.017

**Table 4:** Estimated conditional average treatment effects for subgroups discovered by the causal forest algorithm.

Preschool Children		School-Age Children	
Subgroups	CATC	Subgroups	CATC
Mother’s education: primary school	0.214 ** (0.174)	Mother’s education: no formal education	−3.650 (1.290)
Living area: rural area	0.118 ** (0.090)	Living area: rural area	−1.835 (0.542)
Living area: urban area	0.140 (0.075)	Living area: urban area	0.285 * (0.369)
Child’s gender: male	0.098 ** (0.236)	Child’s gender: male	−1.410 (0.610)
Child’s gender: female	0.139 (0.092)	Child’s gender: female	−0.423 (0.345)
Geographic location: central region	0.205 (0.106)	Geographic location: western region	−2.030 (1.030)
Wealth quantile 2	0.218 * (0.152)	Wealth quantile 2	−0.726 (0.568)
Wealth quantile 5	0.160 (0.097)	Wealth quantile 4	−1.243 (0.519)

Note: *, and ** indicate that the coefficients significantly differ from 0 at the 10%, and 5% levels.

## Data Availability

Publicly available datasets were analyzed in this study. This data can be found here: http://isss.pku.edu.cn/cfps/download/login (accessed on 13 January 2021).

## References

[1] Lei X., Lin W. (2009). The new cooperative medical scheme in rural China: Does more coverage mean more service and better health?. Health Econ..

[2] Cheng L., Liu H., Zhang Y., Shen K., Zeng Y. (2015). The impact of health insurance on health out-comes and spending of the elderly: Evidence from China’s New Cooperative Medical Scheme. Health Econ..

[3] Sun Y. (2019). Welfare consequences of access to health insurance for rural households: Evidence from the New Cooperative Medical Scheme in China. Health Econ..

[4] Shao H.K., Sheng J. (2013). The effect of national health insurance on mortality and the SES—Health gradient: Evidence from the elderly in Taiwan. Health Econ..

[5] Petticrew M., Tugwell P., Kristjansson E., Oliver S., Ueffing E., Welch V. (2011). Damned if you do, damned if you don’t: Subgroup analysis and equity. J. Epidemiol. Community Health.

[6] Hainmueller J., Mummolo J., Xu Y. (2019). How much should we trust estimates from multiplicative interaction models? Simple tools to improve empirical practice. Political Anal..

[7] Athey S., Imbens G. (2016). Recursive partitioning for heterogeneous causal effects. Proc. Natl. Acad. Sci. USA.

[8] Hanratty M. (1996). Canadian national health insurance and infant health. Am. Econ. Rev..

[9] Currie J., Gruber J. (1996). Health insurance eligibility, utilization of medical care and child health. Q. J. Econ..

[10] Currie J., Gruber J. (1996). Saving babies: The efficacy and cost of recent changes in the Medicaid eligibility of pregnant women. J. Political Econ..

[11] Currie J., Gruber J. (1997). The technology of birth: Health insurance, medical interventions and infant health. Working Paper 5985.

[12] Currie J., Decker S., Lin W. (2008). Has public health insurance for older children reduced disparities in access to care and health outcomes?. J. Health Econ..

[13] Wagstaff A., Pradhan M. (2005). Health insurance impacts on health and nonmedical consumption in a developing country. World Bank Policy Research Working Paper.

[14] Chou S.Y., Grossman M., Liu J.T. (2014). The impact of national health insurance on birth outcomes: A natural experiment in Taiwan. J. Dev. Econ..

[15] Chen Y., Jin G.Z. (2012). Does health insurance coverage lead to better health and educational out-comes? Evidence from rural China. J. Health Econ..

[16] Alcaraz C., Chiquiar D., Orraca M.J., Salcedo A. (2017). The effect of Publicly Provided Health In-surance on Education Outcomes in Mexico. World Bank Econ. Rev..

[17] Bagnoli L. (2019). Does health insurance improve health for all? Heterogeneous effects on children in Ghana. World Dev..

[18] Athey S., Imbens G. (2017). The state of applied econometrics: Causality and policy evaluation. J. Econ. Perspect..

[19] Wager S., Athey S. (2018). Estimation and inference of heterogeneous treatment effects using random forests. J. Am. Stat. Assoc..

[20] Chin S., Kahn M.E., Moon H.R. (2020). Estimating the gains from new rail transit investment: A ma-chine learning tree approach. Real Estate Econ..

[21] Andrew J.T. (2019). Machine Learning and Causality: The Impact of Financial Crises on Growth.

[22] Noemi K., Andrew M., Rodrigo M.S., Taufik H., Karla D., Marc S. (2020). Who benefits from health insurance? Uncovering heterogeneous policy impacts using causal machine learning. Working Paper. CHE Research Paper.

[23] Rosenbaum P.R., Rubin D.B. (1983). The central role of the propensity score in observational studies for causal effects. Biometrika.

[24] Robinson P.M. (1988). Root-N-consistent semiparametric regression. Econom. Soc..

[25] Chernozhukov V., Chetverikov D., Demirer M., Duflo E., Hansen C., Newey W., Robins J. (2018). Double/debiased machine learning for treatment and structural parameters. Econom. J..

[26] Athey S., Tibshirani J., Wager S. (2019). Generalized random forests. Ann. Stat..

[27] Breiman L. (2001). Random forests. Mach. Learn..

[28] Meng Q., Chi W.S., Ran T. (2020). BitCoin: A New Basket for Eggs. Econ. Model..

[29] Su C.W., Qin M., Tao R., Umar M. (2020). Financial implications of fourth industrial revolution: Can bitcoin improve prospects of energy investment?. Technol. Forecast. Soc. Chang..

[30] Tibshirani J., Athey S., Wager S., Friedberg R., Miner L., Wright M. (2018). Grf: Generalized Ran-dom Forests (Beta). https://grf-labs.github.io/grf.

[31] Athey S., Wager S. (2019). Estimating treatment effects with causal forests: An application. Obs. Stud..

[32] Chernozhukov V., Demirer M., Duflo E., Fernandez-Val I. (2018). Generic machine learning inference on heterogeneous treatment effects in randomized experiments. Working Paper 24678.

[33] Nie X., Wager S. (2021). Quasi-oracle estimation of heterogeneous treatment effects. Biometrika.

